# Abortion care availability, readiness, and access: linking population and health facility data in Kinshasa and Kongo Central, DRC

**DOI:** 10.1186/s12913-023-09647-6

**Published:** 2023-06-20

**Authors:** Sophia Magalona, Haley L. Thomas, Pierre Z. Akilimali, Dynah Kayembe, Caroline Moreau, Suzanne O. Bell

**Affiliations:** 1 Department of Population Family and Reproductive Health, Johns Hopkins Bloomberg School of Public Health, 615 N. Wolfe Street, Baltimore, MD 21205 USA; 2 Department of Biostatistics and Epidemiology, Kinshasa School of Public Health, University of Kinshasa, Kinshasa, Democratic Republic of Congo; 3 Performance Monitoring for Action DRC, Kinshasa, Democratic Republic of Congo; 4 Centre for Research in Epidemiology and Population Health, Institut National de la Santé et de la Recherche Médicale, Villejuif, France

**Keywords:** Abortion, Postabortion care, Democratic Republic of Congo, Facility data, Survey

## Abstract

**Background:**

The Democratic Republic of Congo (DRC) legalized abortion in 2018 to preserve health and pledged to provide quality postabortion care (PAC), yet little is known about the availability of abortion care services and if facilities are prepared to provide them; even less is known about the accessibility of these services. Using facility and population-based data in Kinshasa and Kongo Central, this study examined the availability of abortion services, readiness of facilities to provide them, and inequities in access.

**Methods:**

Data on 153 facilities from the 2017–2018 DRC Demographic and Health Survey Service Provision Assessment (SPA) were used to examine signal functions and readiness of facilities to provide services across three abortion care domains (termination of pregnancy, basic treatment of abortion complications, and comprehensive treatment of abortion complications). To examine PAC and medication abortion provision before and after abortion decriminalization, we compared estimates from the 2017–2018 SPA facilities to estimates from the Performance Monitoring for Action (PMA) data collected in 2021 (n = 388). Lastly, we assessed proximity to PAC and medication abortion using PMA by geospatially linking facilities to representative samples of 2,326 and 1,856 women in Kinshasa and Kongo Central, respectively.

**Results:**

Few facilities had all the signal functions under each abortion care domain, but most facilities had many of the signal functions: overall readiness scores were > 60% for each domain. In general, readiness was higher among referral facilities compared to primary facilities. The main barriers to facility readiness were stock shortages of misoprostol, injectable antibiotics, and contraception. Overall, provision of services was higher post-decriminalization. Access to facilities providing PAC and medication abortion was almost universal in urban Kinshasa, but patterns in rural Kongo Central showed a positive association with education attainment and wealth.

**Conclusion:**

Most facilities had many of the necessary signal functions to provide abortion services, but the majority experienced challenges with commodity availability. Inequities in accessibility of services also existed. Interventions that address supply chain challenges may improve facility readiness to provide abortion care services, and further efforts are needed to narrow the gap in accessibility, especially among poor women from rural settings.

## Background

Unsafe abortion continues to be a leading cause of maternal mortality and morbidity, particularly where abortion is legally restricted, attributable to 4.7–13.2% of maternal deaths globally each year [1]. Almost half (45%) of abortions worldwide are considered unsafe, meaning the procedure was not conducted by a trained provider using recommended methods [2]. Unsafe abortions are especially high in low-resource settings. In sub-Saharan Africa (SSA), about three quarters of abortions (77%) are unsafe, contributing to an annual case-fatality rate of 185 maternal deaths per 100,000 abortions, which is higher than any other region and at least ten-fold higher than the rate in high-resource settings [3]. Heterogeneity in case-fatality rates is a result of differences in healthcare infrastructure and specifically the availability of safe abortion care (SAC) [4].

SAC is an essential component of sexual and reproductive health services, enabling women to safely terminate unwanted pregnancies and treat complications that may arise from unsafe abortion. While most countries in SSA have highly or moderately restrictive abortion laws that either prohibit or only allow the procedure to save a woman’s life [2], all have pledged to provide quality postabortion care (PAC) – a component of SAC to treat unsafe abortion complications – as part of the 1994 International Conference on Population and Development [5]. PAC services can address incomplete abortions, treat life-threatening complications, and provide postabortion contraception to help prevent future unwanted pregnancies [3, 6]. In SSA, PAC services have expanded in many countries in recent years, yet provision remains inadequate [7–11] and service quality greatly varies across settings [12].

Evaluating health systems’ ability to provide safe abortion and PAC must go beyond an assessment of the service availability to include an evaluation of service-specific readiness to deliver *quality* care. The first efforts to measure service-specific readiness were developed by the United Nations (UN) via signal functions for Emergency Obstetric Care - a set of structural and process indicators intended to measure the ability to provide different levels of care to properly address obstetric complications [13]. Healy and colleagues (2006) first applied the signal functions framework to SAC, highlighting elements necessary for facilities to provide basic SAC at the primary level and additional elements required for comprehensive SAC at the referral level [14]. Basic SAC consists of safe abortion and PAC services for pregnancies less than 12 weeks’ gestation in addition to postabortion contraception, and comprehensive SAC consists of all basic SAC functions for pregnancies more than 12 weeks’ gestation as well as provision of blood transfusions and major abdominal surgery. This model has since been adopted to assess abortion and PAC service readiness in a few sub-Saharan African countries, with studies in general finding low levels of readiness across settings [7, 9–11, 15]. A multi-country analysis by Owolabi et al. found that less than 10% of primary facilities were “ready” (defined as having all signal functions) to provide basic PAC in five out of seven SSA countries; the proportion of referral facilities ready to provide comprehensive PAC was slightly higher, ranging from 23 to 58% [10].

**Table 1:** Dimensions of abortion care and their signal functions

	Termination of Pregnancy	Basic PAC	Comprehensive PAC
Has misoprostol in stock	x		
Has ≥ 1 doctor, degree nurse or degree midwife	x	x	x
Has functioning vacuum aspirator (vacuum aspiration kit or D&C kit)	x	x	x
Has at least one short- and one long-acting reversible contraception in stock	x	x	x
Has obstetric staff present or on-call at all times		x	x
Has injectable antibiotics in stock		x	x
Has injectable uterotonics or misoprostol in stock		x	x
Has intravenous fluid in stock		x	x
Performed blood transfusion in obstetric context in last 3 mos.			x
Performed cesarean section in last 3 mos.			x
Has ≥ 1 doctor			x

In 2018, the Democratic Republic of Congo (DRC) decriminalized abortion by codifying their ratification of the African Union’s Maputo Protocol into law [4]. Abortion is now legal in cases where continuing the pregnancy endangers the mental and physical health of the woman. The Ministry of Health approved the corresponding updated comprehensive abortion care guidelines in 2020, which included allowances for task shifting to mid-level providers at any facility with adequate equipment, thus broadening the cadre of abortion providers and facilities that could offer SAC services. In 2020, the country also took a critical step in expanding access to medication abortion with the inclusion of mifepristone in the essential medicines list; the World Health Organization (WHO) recommends the use of mifepristone and misoprostol (introduced to the list in 2012) as a combined regimen or misoprostol alone for medical abortion [16].

Even before this reform, abortions were common, many of which were unsafe and resulted in complications requiring PAC. Results from the 2016 Kinshasa Abortion Study estimated 146,700 pregnancy terminations in Kinshasa alone in 2016, approximately 34% of which were likely to have involved complications requiring treatment at a health facility [17]. In addition, almost one-fourth of women who had abortions did not receive care from a health provider, while the quality of the PAC the other 77% received was uncertain. Results from a recent study in the DRC assessing national PAC services suggest PAC availability and readiness were low: only 4.7% and 3.8% of facilities in Kinshasa had all basic and all comprehensive PAC signal functions, respectively, while nationally, 3.7% and 1.4% of facilities met these standards and only 13.4% of facilities had misoprostol in stock [15]. The authors were not able to evaluate the geographic accessibility of services, which is important to determine the extent to which clients who may need services are able to reach them. Additionally, availability has not been examined among facilities since the legal reform, though estimates from 2021 suggest abortion incidence is high; 105 per 1,000 women aged 15–49 in Kinshasa and 44 per 1,000 in the rural province of Kongo Central [18]. Approximately one-third and 43% of abortions in Kinshasa and Kongo Central, respectively, involved a non-recommended abortion method and/or source, signaling the continued need for efforts to improve availability of safe termination of pregnancy services and postabortion care for treatment of complications in this post-decriminalization era in the DRC [18]. Examining accessibility in conjunction with the abortion and PAC readiness of the nearby facilities can provide a more complete picture of the abortion landscape available to those experiencing an unintended pregnancy in a specific geography.

The current study seeks to address these gaps and provides a comprehensive examination of the status of abortion services and access in two provinces of the DRC – Kinshasa, the capitol, and Kongo Central, a rural area – using Healy et al.’s (2006) SAC signal functions framework. Using multiple data sources, we aimed to (1) assess SAC availability and facility readiness to provide SAC in each province using a representative sample of facilities, (2) describe PAC and medication abortion provision before and after decriminalization of abortion by facility characteristics, and (3) examine disparities in geographic accessibility of facilities providing PAC and medication abortion across sociodemographic groups by linking facility data to representative samples of reproductive-aged women in Kinshasa and Kongo Central.

**Table 2:** Health facility characteristics in Kinshasa and Kongo Central, SPA 2017–2018

		Kinshasa		Kongo Central	
		n	%*	n	%*
Facility Type					
	Referral	41	19.3	58	36.7
	Primary	32	80.7	22	63.3
Managing Authority					
	Public	23	18.1	40	53.6
	Private	50	81.9	40	46.4
Total		73	100.0	80	100.0

*Weighted

## Methods

### Demographic and Health Survey Service Provision Assessment

#### Survey Overview

We used data from the DRC Demographic and Health Survey (DHS) Service Provision Assessment (SPA) collected from October 2017 to April 2018 to describe facility readiness for our first aim and to describe PAC and medication abortion service provision before the decriminalization of abortion in 2018 for our second aim. The SPA–funded by the United States Agency for International Development, the United States President’s Malaria Initiative, and the Global Fund–is designed to collect information regarding health service availability and delivery within country-wide health systems using five core components: the Inventory, Health Worker Interview, Newborn Resuscitation Simulation, Observation Protocols, and Client Exit Interview questionnaires. The questionnaires were modified for the DRC context by the Ministry of Health (MoH) [19]. Data for this analysis was taken from the Inventory Questionnaire only, which asked about the availability of equipment, commodities and staff in health facilities.

**Table 3:** Percentage of health facilities with all signal functions and readiness scores by abortion care dimension, SPA 2017–2018

	Kinshasa						Kongo Central					
	Termination of Pregnancy (n = 73)		Basic PAC (n = 73)		Comprehensive PAC (n = 41)		Termination of Pregnancy (n = 80)		Basic PAC (n = 80)		Comprehensive PAC (n = 58)	
	% of facilities with all signal functions	Readiness score*	% of facilities with all signal functions	Readiness score*	% of facilities with all signal functions	Readiness score*	% of facilities with all signal functions	Readiness score*	% of facilities with all signal functions	Readiness score*	% of facilities with all signal functions	Readiness score*
Facility type												
Referral	32.7	72.6	20.7	72.5	19.0	77.1	17.9	70.7	19.7	77.6	17.0	83.1
Primary	1.3	46.1	1.2	64.3	--	--	1.0	46.6	1.0	65.6	--	--
Managing Authority												
Public	7.6	68.5	2.4	68.9	2.2	78.9	8.7	66.3	9.5	75.4	24.8	86.2
Private	7.2	57.5	5.5	68.9	24.9	75.7	5.4	61.9	5.9	73.2	12.2	80.6
Total	7.2	61.0	4.9	68.9	19.0	77.1	7.2	64.1	7.9	74.3	17.0	83.1

*Readiness scores range from 0 to 100, percentages weighted

Using a sampling frame of 12,050 health facilities provided by the MoH, probability proportional to size sampling was used to select 1,412 facilities for the SPA. The resulting sample covered all 26 provinces in the country with an average of 50 facilities in each. A total of 1,380 facilities participated in the survey (response rate 98%); the other 32 were in areas of armed conflict at the time of data collection and were therefore not reached. We limited our analysis to facilities in Kinshasa (n = 73) and Kongo Central (n = 80). The survey was administered in French to health providers or facility managers by medical professionals who underwent three weeks of interviewer training. Kinshasa School of Public Health (KSPH) led the data collection in collaboration with the MoH with technical assistance from ICF International.

**Table 4:** Percentage of health facilities with each signal function indicator by facility type, SPA 2017–2018

Abortion care domains**			Signal Function	Kinshasa						Kongo Central					
				Referral		Primary		Total		Referral		Primary		Total	
				n	%*	n	%*	n	%*	n	%*	n	%*	n	%*
**TOP**			Has misoprostol in stock	20	73.3	6	14.7	26	26.8	22	29.5	7	29.3	29	29.3
	**Basic PAC**	**Comprehensive PAC**	Has ≥ 1 doctor, degree nurse or degree midwife	41	100.0	27	83.8	68	86.9	58	100.0	12	52.8	70	70.2
			Has functioning vacuum aspirator (vacuum aspiration kit or D&C kit)	28	73.3	17	47.6	45	52.5	44	72.9	16	71.7	60	72.1
			Has at least one short- and one long-acting reversible contraception in stock	30	69.3	12	34.2	42	41.0	40	49.6	6	24.5	46	33.7
			Has obstetric staff present or on-call at all times	40	99.4	31	96.0	71	96.7	57	98.4	21	95.3	78	96.4
			Has injectable antibiotics in stock	15	61.9	12	34.7	27	40.0	26	37.5	10	43.4	36	41.2
			Has injectable uterotonics or misoprostol in stock	34	91.2	23	67.7	57	72.2	48	69.5	19	85.9	67	79.8
			Has intravenous fluid in stock	20	65.9	22	67.4	42	67.1	42	71.3	17	76.4	59	74.5
			Performed blood transfusion in obstetric context in last 3 months	31	84.4	--	--	31	84.4	54	84.8	--	--	54	84.8
			Performed cesarean section in last 3 months	36	92.6	--	--	36	92.6	55	87.5	--	--	55	87.5
			Has ≥ 1 doctor	41	100.0	--	--	41	100.0	58	100.0	--	--	58	100.0
			Total	41	100.0	32	100.0	73	100.0	58	100.0	22	100.0	80	100.0

*Weighted

**TOP = Termination of pregnancy; PAC = Postabortion care

#### Facility readiness measures

For our first aim—to describe province-specific facility readiness for SAC—we examined three dimensions of abortion care–termination of pregnancy, basic treatment of postabortion complications, and comprehensive treatment of postabortion complications–based on signal function indicators using DHS SPA data as defined by Glover and colleagues, which they adapted from Healy et al.’s framework [14, 15]. The three dimensions of abortion care and the signal functions used to define readiness for each are presented in Table 1. Because primary care facilities in the DRC are expected to provide long-acting reversible contraception (LARC) if they have trained staff, and at minimum SAC requires at least one doctor, degree nurse, or degree midwife for each of the three domains, we modified the signal functions framework by requiring the availability of at least one short-acting method and at least one LARC for each domain. Additionally, it is important to note that the WHO recommends the use of vacuum aspiration for surgical abortion or medication abortion for pregnancies up to 14 weeks and for treatment of postabortion complications; it suggests that these methods replace dilation and curettage (D&C) due to safety issues [16]. However, the SPA Inventory Survey asks about a functioning “vacuum aspiration kit or D&C kit”, and these two methods could not be separated in the data; hence, we included the D&C kit in our signal functions despite it no longer being a recommended termination or PAC method. Each signal function indicator was operationalized as a binary variable to denote availability. Equipment needed to have been observed and recorded as functioning while commodities needed to be observed and with valid expiration date at the time of data collection. For the signal function about contraceptive availability, at least one short-acting method (pills, injections, emergency contraception, and male and female condoms) and at least one LARC (IUDs and implants) had to be available. Services were coded as available if they had been provided in the prior three months. Finally, for staffing, if at least one of the provider types needed for basic SAC (24/7 on-call obstetric staff plus a doctor, degree nurse, or degree midwife) or comprehensive SAC (24/7 on-call obstetric staff plus a doctor) was employed or temporarily employed at the facility, we considered them available. Service provision variables were not included in termination of pregnancy or basic PAC readiness estimates, as other included variables measured the same aspect of care (e.g., having manual vacuum aspiration or misoprostol in stock and performing removal of retained products of conception within the last three months). Service provision variables were included in the comprehensive PAC readiness estimates, however, as those services are not captured in other variables.

**Table 5:** Percentage of health facilities providing PAC, misoprostol, and the combipack before and after abortion decriminalization*

	Kinshasa							Kongo Central						
	n		PAC (%)		Misoprostol (%)		Combipack (%)	n		PAC (%)		Misoprostol (%)		Combipack (%)
	Pre-2018	Post-2018	Pre-2018	Post-2018	Pre-2018	Post-2018	Post-2018	Pre-2018	Post-2018	Pre-2018	Post-2018	Pre-2018	Post-2018	Post-2018
Facility type														
Referral	41	28	74.3	77.7	73.3	54.1	28.7	58	46	54.4	74.3	29.5	57.7	26.4
Primary	32	119	57.6	48.6	14.7	29.6	21.8	22	80	42.5	58.7	29.3	32.3	12.2
Managing Authority														
Public	23	27	57.2	77.3	21.5	63.1	40.4	40	68	29.2	67.9	21.7	35.6	15.5
Private	50	121	61.7	49.1	28.0	28.0	19.3	40	58	67.2	60.4	38.2	48.6	19.7
Total	73	147	60.9	54.2	26.8	34.3	23.1	80	126	46.9	64.4	29.3	41.6	17.4

*Results present the percentage of facilities. Pre-2018 uses DHS 2017–2018 SPA data, Post-2018 uses PMA SDP 2020–2021 data

Facilities were considered “ready” to provide each dimension of abortion care–termination of pregnancy, basic treatment of postabortion complications, or comprehensive treatment of postabortion complications–if all their corresponding signal functions were available: these facilities – categorized as having “all signal functions” –had all four signal functions for termination of pregnancy, all seven for basic treatment of postabortion complications, and all ten for comprehensive treatment of postabortion complications (Table 1). Given that this is an all-or-nothing measure, we also generated an additive index, weighting each signal function equally, for each dimension of abortion care. This index–referred to as a readiness score–is an estimate that captures the average percent of signal functions for each dimension a facility has given its type, managing authority, and province. This score provides a more nuanced measure of abortion care readiness by allowing us to evaluate how close facilities are to being 100% ready to provide the full range of signal functions. Comprehensive PAC readiness was only assessed among referral facilities.

**Table 6:** Percentage of women aged 15–49 living within 3 km (km) of any facility or a health facility offering PAC, misoprostol, or combipack, PMA linked data^*^

	Kinshasa					Kongo Central				
	% of reproductive-age women living within 3 km of any facility or a facility offering each service					% of reproductive-age women living within 3 km of any facility or a facility offering each service				
	*n*	Any facility	PAC	Misoprostol	Combipack	*n*	Any facility	PAC	Misoprostol	Combipack
Age (years)^a^										
15–19	633	100.0	100.0	92.2	91.0	458	86.0	71.9	54.3	38.4
20–29	751	100.0	100.0	89.6	88.3	514	81.4	62.5	47.9	32.3
30–39	543	100.0	100.0	91.2	89.8	508	85.7	67.5	46.3	30.2
40–49	379	100.0	100.0	94.1	91.4	354	86.8	69.7	48.1	33.7
Education										
None	-	-	-	-	-	126	81.9	***44.7***	***26.1***	***10.1***
Primary^b^	129	100.0	100.0	89.7	82.9	521	76.8	***59.6***	***31.3***	***18.1***
Secondary	1689	100.0	100.0	92.6	90.3	1140	88.4	***72.7***	***57.9***	***41.1***
Higher	509	100.0	100.0	91.5	90.6	61	89.8	***88.4***	***80.0***	***72.5***
Wealth tertile										
Poorest	696	100.0	100.0	91.2	87.7	536	67.5	***47.7***	***18.7***	***10.2***
Middle	766	100.0	100.0	91.7	91.0	606	85.8	***63.0***	***39.6***	***17.7***
Wealthiest	865	100.0	100.0	91.4	90.8	706	96.9	***86.7***	***80.1***	***64.8***
Total	2326	100.0	100.0	91.5	89.9	1848	84.7	67.6	49.0	33.5

^a^Missing age data for 21 and 12 respondents from Kinshasa and Kongo Central, respectively

^b^None/Primary education levels combined for Kinshasa only due to sample size

*Bolded and italicized values p < 0.05 using Rao-Scott corrected weighted chi-square statistic

#### Service provision measures

To present provision of PAC and medication abortion for our second aim, facilities were considered to provide PAC if they performed removal of retained products of conception in the three months prior to the survey and to provide misoprostol if misoprostol was in stock at the facility and observed with at least one unexpired misoprostol tablet.

### Performance monitoring for Action Service Delivery Point and female surveys

#### Survey Overview

We used data from the Performance Monitoring for Action (PMA) initiative to describe PAC and medication abortion service provision post-decriminalization of abortion in 2018 for our second aim and to examine accessibility of services in Kinshasa and Kongo Central for our third aim. PMA conducts female, household, and service delivery point (SDP) surveys in the DRC using resident female interviewers who collect information in their communities on reproductive health services and women’s reproductive health history and practices using smartphones. Full details of the PMA initiative, including study and sampling design, questionnaires, and data, are available online [20]. We used cross-sectional female and SDP data from the Kinshasa and Kongo Central Phase 3 surveys collected in December 2021- April 2022. KSPH implemented the survey, with technical support and overall project direction from The Bill & Melinda Gates Institute at the Johns Hopkins Bloomberg School of Public Health.

**Table 7:** Adjusted odds of living within 3 km of a health facility offering PAC, misoprostol, and the combipack among women aged 15–49, PMA 2021–2022*

	Kinshasa						Kongo Central					
	PAC		Misoprostol		Combipack		PAC		Misoprostol		Combipack	
	aOR^a^	95% CI	aOR^a^	95% CI	aOR^a^	95% CI	aOR^a^	95% CI	aOR^a^	95% CI	aOR^a^	95% CI
Age (years)												
< 20	-	(ref)		(ref)								
20–29	-		***0.72***	***(0.52, 0.99)***	0.77	(0.56, 1.06)	***0.67***	***(0.47, 0.95)***	0.82	(0.57, 1.18)	0.78	(0.52, 1.15)
30–39	-		0.87	(0.63, 1.21)	0.89	(0.66, 1.20)	0.98	(0.68, 1.43)	0.91	(0.63, 1.31)	0.84	(0.55, 1.30)
40–49	-		1.37	(0.83, 2.26)	1.11	(0.69, 1.80)	1.06	(0.66, 1.70)	0.92	(0.61, 1.40)	1.00	(0.67, 1.48)
Education												
None	-						(ref)		(ref)		(ref)	
Primary^b^	-		(ref)		(ref)		2.08	(0.91, 4.77)	1.41	(0.51, 3.88)	2.09	(0.66, 6.59)
Secondary	-		1.304	(0.67, 2.52)	1.75	(0.99, 3.09)	2.49	(0.87, 7.13)	2.45	(0.85, 7.10)	***3.71***	***(1.40, 9.84)***
Higher	-		1.419	(0.50, 4.06)	1.83	(0.74, 4.52)	5.10	(0.90, 28.87)	***4.42***	***(1.23, 15.89)***	***9.45***	***(3.28, 27.23)***
Wealth tertile												
Poorest	-		(ref)		(ref)		(ref)		(ref)		(ref)	
Middle	-		1.04	(0.37, 2.9)	1.33	(0.48, 3.74)	1.83	(0.65, 5.12)	***2.69***	***(1.20, 6.03)***	1.77	(0.81, 3.90)
Wealthiest	-		0.95	(0.31, 2.87)	1.24	(0.41, 3.76)	***6.25***	***(1.73, 22.57)***	***14.49***	***(4.42, 47.46)***	***13.22***	***(3.04, 57.42)***

^a^Adjusted odds ratio

^b^None/Primary education levels combined for Kinshasa only due to sample size

*Bolded and italicized values p < 0.05

The female survey used a stratified cluster design to generate representative samples at the provincial level. Samples of 58 and 52 census enumeration areas (EA) were drawn separately from master sampling frames for Kinshasa and Kongo Central, respectively, using probabilities proportional to size. All households within the selected EA were mapped and listed, and a random selection of 33 households within each EA were invited to participate. Household heads completed the household interview, and all resident women aged 15–49 in the selected households were eligible to participate in the face-to-face female survey. Interviews were conducted primarily in French and as needed, local dialects, using agreed upon oral translations. Out of 2,475 and 1,897 women who were eligible to participate in the survey in Kinshasa and Kongo Central, respectively, 2,326 (94.0% response rate) and 1,856 (97.8% response rate) completed the female interviews.

For the SDP survey, sampling aimed to include the public facilities that served each EA and up to three private facilities within the EA boundary. The private facilities were selected from a list generated by interviewers through mapping and listing, and public facilities were identified via a list obtained from local health authorities. Trained interviewers administered the survey to all identified SDPs, which included questions about reproductive health service provision, commodity stock, cost, quality of services, and other related topics. Survey respondents were management staff answering on behalf of the facility. Out of the 229 and 201 SDPs selected for in Kinshasa and Kongo Central, respectively, 197 (86%) and 191 (95%) completed the survey. Although pharmacies (n = 115) provide medication abortion pills, they were excluded from the analysis as they were not asked about postabortion care or misoprostol and the combination therapy of misoprostol and mifepristone (hereinafter referred to as combipack) availability in the PMA survey. The resulting analytical sample included 273 facilities (n = 147 in Kinshasa, n = 126 in Kongo Central).

#### Service provision measures

To capture provision of services, SDP survey respondents were asked “Which of the following services are provided at this facility?”. Facilities were considered to provide PAC if they selected “Postabortion services” from the answer options. For misoprostol and the combipack, respondents were asked “Is Miso-Kare (misoprostol) available in the facility for management of postpartum hemorrhage or other gynecologic issues?” and “Is Ma-Kare (misoprostol & mifepristone) available in the facility?”, respectively, and responded “yes” or “no”. If the medications were reported as available, interviewers recorded whether they were “in-stock and observed”, “in-stock but not observed”, or “out of stock.” A facility was considered to provide misoprostol or the combipack if the medication was in-stock and observed.

#### Accessibility measures

We linked the SDP and female samples using geospatial data to examine the percentage of women who have access to facilities providing abortion services. Global Positioning System points were taken at the time of each SDP and female interview, and each woman’s distance to any sampled facility and to a facility that provided PAC, misoprostol, or the combipack was calculated in kilometers using Euclidean distance, a method applied in a similar study exploring accessibility of services [9]. Four access variables were developed, assigning either 0 or 1 to each woman based on whether she had access—defined as being within a three km distance—to any facility or a facility providing PAC, misoprostol, or the combipack.

Facility type was recorded differently between the two data sources. To keep categories uniform across the two data sources, the facilities were grouped into: (a) referral facilities, which included tertiary/provincial hospital, general reference hospital, hospital/clinic, and reference health center categories in the SPA and hospital, health clinic, and other in the SDP; and (b) primary facilities, which include health center in the SPA and health center and health post in the SDP. Facilities were also grouped by management authority (public or private). Public facilities included those managed by the government, whereas private facilities were managed by non-governmental organizations, for-profit/private organizations, or faith-based organizations.

### Analysis

Our first aim was to describe abortion service readiness in representative samples of DHS SPA facilities in Kinshasa and Kongo Central. We first examined distributions of facility characteristics (facility type and managing authority) in each province. We estimated the percentage of facilities ready to provide each of the signal functions, overall and by facility type in each province. We then calculated the percentage of facilities ready to provide all the signal functions under each dimension and generated readiness scores for termination of pregnancy, basic, and comprehensive care separately, by facility type and managing authority in each province. While referral and primary facilities were included in the denominator for both readiness measures of the termination of pregnancy and basic PAC domains, only referral facilities were included in the calculations for comprehensive PAC. The descriptive analyses were weighted to account for the probability of selection and non-response.

Our second aim was to examine PAC and medication abortion provision before and after the decriminalization of abortion in the DRC in 2018. We used the SPA data to describe the proportion of facilities providing PAC, misoprostol, and the combipack before decriminalization and the SDP data for after decriminalization. We applied post-stratification weights to the SDP data to reflect the representative distribution of facilities in the SPA within each province. Provision of the combipack was only calculated using the SDP data, because information about mifepristone was not collected in the SPA. Proportions were presented by facility type and managing authority for each province.

Our third aim was to examine accessibility in each province using the linked PMA data. We described the proportion of women living within three kilometers of facilities that provided PAC, misoprostol, and the combipack by sociodemographic characteristics (age, education, and wealth) separately by province. Sociodemographic characteristics were selected to explore potential relationships with access to services. We then ran two multivariable logistic regression models to assess sociodemographic characteristics associated with access to each service, one model for Kinshasa and another for Kongo Central. In Kinshasa, due to the almost universal access to PAC, regression models were run for only misoprostol and combipack accessibility. The analysis was weighted to account for the complex survey design and clustering in the female data.

The analyses were conducted in Stata version 16 [21].

## Results

### Signal functions and facility readiness to provide abortion services

There were 73 facilities in Kinshasa and 80 facilities in Kongo Central in the SPA (Table 2). The majority of facilities were primary level in both Kinshasa (80.7%) and Kongo Central (63.3%). Most facilities in Kinshasa were private (81.9%), while less than half (46.4%) were private in Kongo Central.

In both provinces, the percent of facilities with all signal functions to provide pregnancy termination, basic PAC, and comprehensive PAC was low, ranging from 4.9 to 19.0% (Table 3). In Kinshasa, 7.2% of facilities had all signal functions for pregnancy termination, 4.9% for basic PAC, and 19.0% for comprehensive PAC. Under pregnancy termination and basic PAC, a higher proportion of referral facilities had all signal function components compared to primary facilities. In Kongo Central, 7.2% had all signal functions for pregnancy termination, 7.9% for basic PAC, and 17.0% for comprehensive PAC. Like Kinshasa, referral facilities were more ready (having all the signal function components) than primary facilities under pregnancy termination and basic PAC. Interestingly, basic PAC was slightly higher in Kongo Central than Kinshasa (7.9% vs. 4.9%), but the opposite was observed for comprehensive PAC (19.0% in Kinshasa vs. 17.0% in Kongo Central).

Despite few facilities in both provinces having all signal function components for the different domains of abortion care, many had several components, as indicated by the readiness scores (Table 3). In Kinshasa, termination of pregnancy had the lowest readiness score at 61.0% of all facilities, followed by basic PAC (68.9% of all facilities); the readiness score was highest for comprehensive PAC (77.1%), though this was only calculated among referral facilities. In Kongo Central, we observed the same pattern -- 64.1% for termination of pregnancy, 74.3% for basic PAC, and 83.1% for comprehensive PAC. Readiness scores were thus higher across all three abortion care domains for Kongo Central compared to Kinshasa. In Kongo Central, the same pattern was observed (83.1% for comprehensive PAC, 74.3% for basic PAC, and 64.1% for termination of pregnancy). Readiness scores were higher across all three abortion care domains in Kongo Central compared to Kinshasa. In both provinces, slightly higher readiness scores were observed for public versus private facilities across all domains, except for basic PAC in Kinshasa. Scores were also higher among referral facilities as compared to primary facilities in both provinces for termination of pregnancy and basic PAC.

Certain signal functions were consistently less available within each of the domains of abortion care (Table 4). Overall, in both Kinshasa and Kongo Central, misoprostol was the signal function most missing when assessing readiness to provide pregnancy termination. The most missing signal functions for basic PAC were injectable antibiotics in Kinshasa and having at least one short- and one LARC in stock in Kongo Central. For comprehensive PAC, facilities in both provinces were often missing blood transfusion.

#### PAC and medication abortion before and after abortion decriminalization

Differences in the provision of PAC and misoprostol before and after abortion was decriminalized in 2018 were observed in both Kinshasa and Kongo Central (Table 5). In Kinshasa, PAC provision post-decriminalization was only slightly higher in referral facilities, but lower by 9% in primary facilities compared to pre-2018. By managing authority, PAC was higher by 20% in public facilities, but lower by almost 13% in private facilities. For misoprostol, provision was lower in referral facilities by almost 20%, but was double in primary facilities after 2018 (14.7% vs. 29.6%). Misoprostol provision in private facilities was almost the same pre- and post-2018, but in public facilities, the proportion post-2018was three-times that of pre-2018 (21.5% vs. 63.1%). In Kongo Central, PAC provision was higher by 16–20% in both referral and primary facilities after 2018. Provision was also more than double in public facilities but lower by about 7% in private facilities. Misoprostol provision was higher in all facility types and managing authorities by 3–28%. Post-decriminalization, the combipack was provided by at least one out of four referral facilities in both provinces, but in primary facilities, only 21.8% provided it in Kinshasa and 12.2% in Kongo Central. In Kinshasa, more than double (40.4%) the public facilities were providing the combipack compared to private facilities (19.3%), whereas in Kongo Central, slightly more private facilities (19.7%) provided it than public facilities (15.5%).

#### Access to abortion services

The PMA SDP sample included 37 (25.2%) referral and 110 (74.8%) primary facilities in Kinshasa, and 22 (17.5%) referral and 104 (82.5%) primary facilities in Kongo Central (results not shown in tables). In Kinshasa, most of the facilities in the sample were private (83.0%), whereas in Kongo Central, slightly over half were public (55.6%).

Access to facilities that provided PAC, misoprostol, and the combipack was almost universal among women in Kinshasa, but more variable in Kongo Central across women of different sociodemographic characteristics (Table 6). In Kinshasa, all women lived within three kilometers of a facility that provided PAC, and over 89% had access to facilities that provided misoprostol. Overall, nine out of ten women (89.9%) lived near a facility that provided the combipack. In Kongo Central, access was more variable, with only two in three women living close to a facility providing PAC (67.6%), half (49.0%) living near a facility providing misoprostol, and one in three (33.5%) living near a facility providing the combipack. PAC access increased as education attainment and wealth increased (44.7% of women who had never attended school to 88.4% of women with higher education; 47.7% of the poorest women to 86.7% of the wealthiest). Similarly, proportions of women who were in proximity to facilities that provided misoprostol and the combipack increased as education attainment and wealth increased.

Results for the multivariate logistic regression models of PAC, misoprostol, and combipack accessibility in Kinshasa and Kongo Central are presented in Table 7. There was no heterogeneity in PAC access in Kinshasa since all women lived within three kilometers of a facility providing the service, thus we omitted those results. For misoprostol, age was associated with access in the capitol province: women aged 20–29 had lower odds (adjust odds ratio (aOR): 0.72, 95% CI: 0.52–0.99) of living near a facility providing misoprostol than women aged < 20 years. Education and wealth were not significantly associated with living near a facility providing misoprostol or the combipack. In rural Kongo Central, we observed more variability in access by sociodemographic groups for all three services, although some confidence intervals were very wide. Age was only significantly associated with access to PAC, with women aged 20–29 years having 0.67 (95% CI: 0.47–0.95) lower adjusted odds of living near a facility providing PAC. Compared to women who had never attended school, women who had higher education had more than four times adjusted odds of living near a facility that provided misoprostol (aOR: 4.42, 95% CI: 1.23–15.89); those with secondary and higher education had three to nine times odds of living near a facility that provided the combipack (secondary education aOR: 3.71, 95% CI: 1.40–9.84; higher education aOR: 9.45, 95% CI: 3.28–27.23). Wealth was also associated with access to all three services, with the wealthiest women having six to fourteen times greater odds of living near a facility providing PAC (aOR: 6.25, 95% CI: 1.73–22.57), misoprostol (aOR: 14.49, 95% CI: 4.42–47.46), and combipack (aOR: 13.22, 95% CI: 3.04–57.42) than the poorest women. Women in the middle wealth group had more than two times odds of living near a facility providing misoprostol (aOR: 2.69, 95% CI: 1.20–6.03) compared to the poorest women.

## Discussion

Our findings show that, before the legal reform for access to safe abortion in the DRC in 2018, readiness to provide abortion services – defined by having all the signal functions for each domain – was low in both Kinshasa and Kongo Central; however, most facilities had many of the signal functions as indicated by their readiness scores. Facilities mostly faced stock shortages of misoprostol, injectable antibiotics, and contraception. These shortages were more pronounced among primary facilities compared to referral facilities in both provinces. Comparing SPA data with more recent data from the PMA initiative to examine differences in service provision after the abortion reform, PAC and misoprostol provision was higher across facility types and sectors in Kongo Central, but only among public facilities in Kinshasa. Finally, while women’s access to PAC and medication abortion was almost universal in the capitol province after the reform, disparities existed in rural areas, with access decreasing as education attainment and wealth decreased.

The low levels of facility readiness for the three abortion service domains are consistent with national findings from Glover and colleagues [15]. Like the results from the national study, the limiting factor in achieving full facility readiness to provide safe abortion care in Kinshasa and Kongo Central is primarily stockouts of commodities—injectable antibiotics, misoprostol, and contraception. A study of contraceptive availability in Kinshasa showed rampant stockouts that were relatively unchanged between 2014 and 2016 [22], signaling a persistent challenge in the supply environment. To minimize this limitation in the health system, investments in solutions focusing on ensuring a well-functioning supply chain are needed. The shortage of misoprostol stock may also be related to its relatively recent introduction to the country’s essential medicines list in 2012 –only five years prior to the SPA data collection—combined with supply chain challenges. Recent introduction may also explain the low levels of combipack provision, given that mifepristone was not registered in the DRC until 2020. Because both medications were only recently introduced and given the changes in abortion legality in the country, health providers may not have had adequate training or be willing to provide these services [15]. The low provision of medication abortion is especially pronounced in rural areas than the capitol province, and our accessibility results pointed to a greater need among poor women and those of lower education attainment who were least likely to live near a facility offering these services. Improving accessibility to SAC services for poor, rural women is likely to improve reproductive health as evidence suggests these populations are most likely to suffer the negative sequelae associated with unsafe abortion and least likely to access treatment for unsafe abortion complications [23].

Expanding on previous studies describing abortion service availability and facility readiness in the DRC, this study is the first to present a comprehensive analysis of abortion service availability, readiness, and accessibility in Kinshasa and Kongo Central, including data collected before and after the liberalization of the abortion law [15]. By using the DHS SPA data, we were able to assess facility readiness to provide abortion services using a representative sample of facilities in both provinces, highlighting individual signal functions that are most often not available and required improvements in supply chains. In addition, the inclusion of two readiness measures enabled us to examine facility readiness to provide quality care in different ways –whether facilities were fully ready versus how ready they were along a continuous spectrum, the latter providing a more nuanced assessment of readiness. Furthermore, using the representative sample of facilities in the DHS SPA, we were able to adjust the more recent SDP sample to reflect the distribution in the health care system and assess differences in the provision of services after the DRC made policy changes for access to safe abortion, demonstrating which facility type, sector, and province experienced improvements over time. Lastly, by linking facility data to a representative sample of women, we were able to evaluate disparities in abortion service access, as defined by living in proximity to a facility providing PAC and medication abortion, among different sociodemographic groups.

However, this study is not without limitations. First, not all signal function indicators were completely aligned with the data captured in the SPA survey. Having a functioning vacuum aspirator, for example–which is recommended by the WHO for surgical abortion of pregnancies up to 14 weeks and for treatment of postabortion complications–was not measured on its own in the Inventory survey; instead, it was combined with the D&C kit, which is no longer recommended by the WHO due to issues with safety [16]. However, results from the 2016 Kinshasa Abortion Study Health Professionals Survey found that, contrary to best practices, D&C was performed by health professionals to terminate pregnancies and was the most common treatment for women receiving postabortion care [24]. Therefore, if D&C kits are still common in facilities, our measure may be higher than if we captured vacuum aspirators alone. Additionally, the SPA Inventory did not collect any information on mifepristone stock at facilities despite it being added to the WHO essential medicines list in 2005 after WHO guidelines recommended the use of the combination regimen (mifepristone and misoprostol) for medication abortion [25]. Mifepristone was registered in the DRC more than two years after the 2017–2018 DHS SPA and so was not included in the survey, therefore we were only able to look at the availability of misoprostol and not the combipack in the SPA. The SPA survey also collected information on the number of health providers at different occupational levels yet did not include any data on specific abortion care training for these positions. This restricted our ability to assess staff readiness based only on the presence of occupational categories at facilities and not on the capacity of staff to perform the necessary abortion procedures. Lastly, the SPA dataset only enabled us to examine signal functions and facility readiness right before the codification of the Maputo Protocol in 2018. Ideally, we would have used the PMA data for a more recent status of these measures, but the data were not collected in the SDP survey.

Additional limitations were present in the PMA data. First, our analysis did not include pharmacies, because they were not asked abortion-service-related questions. We may have eliminated a major source of medication abortion with their exclusion, as findings from the Kinshasa Abortion Study confirmed that women who relied on medication abortion often obtained them from pharmacies [24]. Second, we analyzed access to PAC or medication abortion using proximity to a facility, defined as less than or equal to three kilometers, but our narrow definition of access excludes other factors that may influence women’s access to services, such as costs, transportation availability, and knowledge of services provided. In addition, women may not necessarily seek services at the nearest facility, opting instead for farther facilities that offer better service quality. Studies evaluating women’s facility choice found that they consider many factors when deciding where to go for healthcare, including health provider competency and bias, availability of supplies, level of discretion afforded, and cost of services [26–29]. Finally, there are limitations with the selection of facilities for the SDP survey. Selection only included up to three private facilities in each EA and the public facilities that are assigned to serve that EA, so we may have failed to include nearby facilities that either were outside of the EA boundary or did not serve that EA but may have been less than three kilometers from some women in the sample. In addition, selection was not based on a facility sampling frame. Instead, facilities were selected if they served the nationally representative sample of women for the female questionnaire. Because women were selected using probability proportional to size sampling, facilities that served larger population were more likely to be selected.

## Conclusion

Our findings highlight specific areas that can be targeted by interventions aiming to improve facility provision of and access to abortion services in the DRC. Facilities have many of the components needed to provide safe abortion care services, but shortages in commodities limit their ability to be fully ready to provide services. Supply chain evaluations should be considered to better understand the low levels of these commodities and offer solutions to improve their availability across all facility levels that are supposed to provide services. While there was an overall improvement in the provision of services, particularly in Kongo Central, after the Maputo Protocol was codified into law, our accessibility analysis showed disparities in access across sociodemographic groups. Efforts to improve delivery of abortion services should focus on narrowing the gap in access, particularly trying to understand barriers among disadvantaged groups in rural areas.

## Data Availability

PMA data and materials for this study will be posted publicly at pmadata.org. Once available online, anyone can access after completing a brief request form at https://www.pmadata.org/data/available-datasets. DHS data are available online at https://dhsprogram.com/data/available-datasets.cfm.

## List of Abbreviations

D&C: Dilation and curettage
DHS: Demographic and Health Survey
DRC: Democratic Republic of Congo
EA: Enumeration area
KSPH: Kinshasa School of Public Health
LARC: Long-acting reversible contraceptive method
MoH: Ministry of Health
PAC: Post-abortion care
PMA: Performance Monitoring for Action
SAC: Safe abortion care
SDP: Service delivery point
SPA: Service Provision Assessment
SSA: Sub-Saharan Africa
UN: United Nations
WHO: World Health Organization
